# When accountability meets power: realizing sexual and reproductive health and rights

**DOI:** 10.1186/s12939-020-01221-4

**Published:** 2020-07-08

**Authors:** Gita Sen, Aditi Iyer, Sreeparna Chattopadhyay, Rajat Khosla

**Affiliations:** 1 Distinguished Professor and Director, Ramalingaswami Centre on Equity and Social Determinants of Health, Public Health Foundation of India, Bangalore, India; 2 Senior Research Scientist, Ramalingaswami Centre on Equity and Social Determinants of Health, Public Health Foundation of India, Bangalore, India; 3 Independent Researcher, Amsterdam, Netherlands; 4 Human Rights Advisor for the Human Reproduction Programme at the World Health Organization, Geneva, Switzerland

**Keywords:** Power, accountability, sexual and reproductive health and rights, Measurement, Status quo, Accommodation, Subversion, Contestation, Negotiation, Transformation

## Abstract

This paper addresses a critical concern in realizing sexual and reproductive health and rights through policies and programs – the relationship between power and accountability. We examine accountability strategies for sexual and reproductive health and rights through the lens of power so that we might better understand and assess their actual working. Power often derives from deep structural inequalities, but also seeps into norms and beliefs, into what we ‘know’ as truth, and what we believe about the world and about ourselves within it. Power legitimizes hierarchy and authority, and manufactures consent. Its capillary action causes it to spread into every corner and social extremity, but also sets up the possibility of challenge and contestation.

Using illustrative examples, we show that in some contexts accountability strategies may confront and transform adverse power relationships. In other contexts, power relations may be more resistant to change, giving rise to contestation, accommodation, negotiation or even subversion of the goals of accountability strategies. This raises an important question about measurement. How is one to assess the achievements of accountability strategies, given the shifting sands on which they are implemented?

We argue that power-focused realist evaluations are needed that address four sets of questions about: i) the dimensions and sources of power that an accountability strategy confronts; ii) how power is built into the artefacts of the strategy – its objectives, rules, procedures, financing methods inter alia; iii) what incentives, disincentives and norms for behavior are set up by the interplay of the above; and iv) their consequences for the outcomes of the accountability strategy. We illustrate this approach through examples of performance, social and legal accountability strategies.

## Introduction

This paper addresses a critical concern in *realizing* sexual and reproductive health and rights (SRHR) through policies and programs – the relationship between power and accountability. As the decades have passed since the United Nations International Conference on Population and Development (ICPD) in Cairo in 1994, and the Fourth World Conference on Women in Beijing the following year, the challenge of accountability has continued to loom large in national and global policy spaces. For some portions of the ICPD agenda such as maternal health, accountability to strengthen the roles of duty-bearers as well as rights-holders has come increasingly to the center of policy debates, e.g., the work of the International Accountability Panel (IAP) of the UN Secretary General’s Initiative, *Every Woman, Every Child, Every Adolescent* (https://iapewec.org/). But accountability for other issues has been more contentious and the realization of rights remains painfully hit or miss. This was noted by the High-Level Taskforce (HLTF) for ICPD Beyond 2014 (https://www.icpdtaskforce.org/).

The paper is part of an ongoing response to this challenge, drawing on and going beyond various resources already in place. Previous research on accountability within health systems [1] was mainly concerned to improve health system functioning by reducing patient abuse and ensuring that procedures and standards are met. While this is unexceptionable, respecting, protecting and fulfilling human rights did not feature front and center. More recent SRHR-linked accountability research has advanced our understanding of the human rights challenge at the heart of accountability through a systematic review of the literature [2], and by delineating key elements of context [3]. The systematic review [2] identified three main types of accountability strategies for SRHR: performance, legal and social. These strategies are embedded within key aspects of context - political and moral economies, gendered norms, and barriers to claiming rights [3].

In this paper, we go deeper by examining these three types of accountability strategies (performance, legal and social) through the lens of power so that we might better understand and assess their actual working. Why power? Because, we argue, power relations shape the realization of human rights. While the normative frames for human rights may be well defined, their actual fulfilment depends on power relations that operate at many levels. The interrogation of power must, therefore, be central to understanding whether and how accountability strategies work, and how to assess them. This holds *a fortiori* for sexual and reproductive rights, which are deeply imbricated in power relations from the level of households to the negotiation chambers of the United Nations.

For accountability strategies to meet their aims of increasing transparency, representation, inclusiveness, and responsiveness in SRHR policies and programs, we must acknowledge and engage with power relations that constitute the warp and weft of all societies. In this paper, using illustrative examples, we show that in some contexts accountability strategies may confront and transform adverse power relationships. In other contexts, power may not only give rise to contestation, but possibly accommodation, negotiation or even subversion of the goals of accountability strategies.

The ability of weak states and weak health systems to deliver on accountability goals is a challenge in many contexts [4]. However, we do not address this issue in the paper for reasons of space.

### Conceptualizing power

The definition and study of power is large and contested. A growing conceptual and empirical literature [5–7] evidences increasing recognition of the role of power in health policy and systems. As Erasmus and Gilson [8] state, “… practices of power are at the heart of every policy process …” , and especially visible at what they call “the coalface of implementation”. Policy concern about inequality has fueled evidence that access to power in health is unequal within societies and across them. Funding, health information, and policy directions in global health play a critical role in defining who is powerful and how [9, 10]. Not only bilateral and multilateral funders but also private corporations have come to have increasing say over health policy directions. Uniquely perhaps in the SRHR field, religious institutions and related actors have begun to wield enormous influence in relation to laws, policies and agreements.

Social science literature has examined power relations from many angles that can be helpful to understanding these tendencies in the context of SRHR. They include the structures of power inherent in political economic systems based on unequal control over productive assets, social and cultural capital [11, 12]. These forms of capital underpin sexual and reproductive practices and behaviors by defining the fallback positions of different social groups [13], governing who is dominant or subordinate and why. Feminists and other social movements have emphasized structural inequalities based on gender, ethnicity, caste and other ascriptions, as sources of power in sexuality and reproduction that are deeply imprinted in societies. Considerable evidence worldwide has established that power rooted in such intersecting structural inequalities can have serious consequences for people’s health and specifically for SRHR [14–27].

But structural inequalities and their intersections are not all. The ‘power-knowledge’ systems of society that govern what is believed to be true, and that define the boundaries of norms, discourses and behaviors are both insidious and powerful. Power can be used to “manufacture consent” [28, 29] and to socialize through education and the media [30–32]. Foucault [33] described the capillary-like reach of power into the deepest and subtlest recesses of society including people’s sense of themselves as subjects or agents. The historical rise of bio-power, “an explosion of numerous and diverse techniques for achieving the subjugations of bodies and the control of populations” ([34], p140), is linked to the diffusion of power throughout society. Bio-power obtains compliance in many ways, ranging from persuasion, bribery, and assertions of authority, to economic or other coercion, and violence or threats of violence [35]. A range of beliefs and practices around sexuality and reproduction - “proper” masculinity and femininity, child marriage, menarche rituals, menstruation practices, do’s and don’ts around sex and childbearing especially among adolescents, people with disabilities or outside marriage, pregnancy and contraception, widowhood, gendered divisions of work and control over economic and other resources such as knowledge and information, heteronormative biases and discrimination, to name only some – provide ample evidence of such methods of obtaining compliance.

However, just as capillary action in the human body often has to push upwards against gravity, the capillarity of power opens the possibility of resistance from the margins – a crucial doorway for thinking about both the challenges to realizing sexual and reproductive rights, and the possibility of accountability. Ordinary people including especially adolescents, youth and women, civil society organizations, or ‘street-level bureaucrats’– frontline actors such as community health workers and other lower level service providers [36] – can demand accountability, challenging and even transforming policies in their efforts to achieve greater control over their work and lives [37].

Feminist scholar-activists have long viewed gender power as an amalgam of structural inequalities and socialization through gendered norms and knowledge systems [24]. The original feminist usage of women’s ‘em-power-ment’, with power at its centre, referred to both extrinsic control over different kinds of resources and intrinsic control over beliefs about self and the world [38–42]. Extending this work specifically to SRHR, Sen and Batliwala ([43], p35) proposed a range of measures to counter gender power and to strengthen accountability for advancing SRHR. The implications for health policy processes and for accountability strategies can be profound.

In sum: power is central to the working of accountability strategies in health policies, and in SRHR specifically. Power often derives from deep structural inequalities, but also seeps into norms and beliefs, into what we ‘know’ as truth, and what we believe about the world and about ourselves within it, legitimizing hierarchy and authority and manufacturing consent. Its capillary action causes it to spread into every corner and social extremity, but also sets up the possibility of challenge and contestation.

### How power works in accountability for SRHR

Tackling different forms of power to advance SRHR through accountability strategies requires acknowledging that dominant power can be challenged by those subjugated by hierarchies and subordination. The workings of power involve not only domination but also forms of resistance and contestation, building on power’s capillarity, and the fact that structural inequalities are not immutable. Accountability strategies can support “empowerment” that can energize and motivate the apparently powerless to action, resulting in a range of processes that are the subject of this section. However, not all such workings of power will support or promote accountability – some may do so, while others may thin down or hollow out the strategy. The best policy and accountability intentions can be waylaid. But realistic expectations can also point to possibilities for transforming the deleterious effects of power, while taking advantage of its weak links.

We use a set of illustrative cases to show how power may work to reinforce a status quo, weaken an accountability strategy, or transform a situation leading to realization of sexual and reproductive rights. The cases were chosen from existing literature to cover all six of the processes / outcomes described below, through a mix of both pragmatic and substantive criteria: how well they were documented in English, whether power relations were clearly illustrated, and whether together they covered a mix of major SRHR themes. The chosen illustrations cover family planning, maternal health (maternal death reviews, and disrespect/abuse), HIV/AIDS, abortion, the functioning of community health workers, and sexual violence in conflict.

The six processes / outcomes illustrated below may not exhaust all possibilities. They are also not mutually exclusive in that the workings of any strategy may involve a mix of them.

#### Reinforcing dominant power relations

Strategies intended to improve *performance accountability* through various artefacts - rules, orders, financing mechanisms, data collection and information use - may end up reinforcing instead of transforming power relations. The artefacts of the strategy may reinforce power relations in ways that are inimical to realizing human rights, even if performance indicators improve. A well-known illustration of this is the use of targets, incentives and disincentives in family planning programs to improve performance accountability [44]. In the pre-ICPD decades, family planning programs were often driven by population control aims without explicit recognition of individual human rights and bodily autonomy / integrity [44]. The power of the state was mobilized vis-a-vis citizens, with frontline health workers and other community level workers expected to carry out the state’s mission of population control. In this context, targets, incentives and disincentives were often explicitly intended to improve the performance of health workers on the ground. But they have been criticized for skewing attention away from other primary health care tasks, prioritizing female sterilization over temporary methods or the provision of choices, and leading to human rights concerns resulting from the pressure to meet targets [44].

#### Accommodation

Programme implementation may appear on the surface to respond to an accountability strategy, while being, in reality, tokenistic and even reinforcing the power status quo. Kapilashrami and McPake’s study [45] of the power dynamics set off by the entry of the Global Fund to Fight AIDS, TB and Malaria (the Fund) in countries uncovers the dissonance between stated intentions and actual processes. Central to the Global Fund’s rules were multiple elements that are usually viewed as promoting participation and *social as well as performance accountability.* These included *country ownership* through leaving implementation to national bodies, *inclusiveness and partnership* through direct funding of civil society as well as a role for them in the country coordination mechanism, and *evidence- and performance-based funding*. Underneath what the authors call this “public transcript” was a “hidden transcript” in which the real power differentials between international funders and national government bodies, between international NGOs and domestic ones, between government officials and civil society were played out. As a result, corners were cut on mandated participation rules and mechanisms in myriad ways. On the surface, all required procedures were accommodated and followed, allowing everyone to claim success. A quite different hidden reality of power struggles, delays in the sharing of information and closed-door negotiations, inter alia*,* epitomized the underlying tensions, and mocked these claims of participation and social / performance accountability.

#### Subversion

Power may completely undermine the intentions of an accountability strategy. In a study of what they call ‘micro-practices’ of power, Lehmann and Gilson [37] show how a national policy to extend and systematize the reach of community health workers (CHWs) ended up doing the opposite. CHWs are often seen as supportive of both *performance and social accountability*, because of their closeness to their communities, and their in-depth knowledge of their needs and constraints. However, in this study in one sub-district, the combination of intentions that were at cross-purposes (providing jobs and stipends to more educated youth rather than to older women CHWs who had long been doing unpaid work in their communities), and the exercise of power by different health managers trying to retain and extend their authority and control ended up with a subversion of the strategy’s core purpose. “At the time of this study the most significant outcome of the policy implementation process had been the reduction or thinning of very complex policy intentions and objectives to a single outcome, namely the payment of stipends to a small number of CHWs. Simultaneously, a large number of mostly older, experienced CHWs had withdrawn from the programme because they were not selected to be paid stipends and felt that their long-standing commitment and contribution had gone unacknowledged. Overall, therefore, all facilities had substantially fewer CHWs available than before the introduction of the national policy.” (37, p361–2).

#### Contestation

Differently from accommodation and subversion that work in more hidden ways, and where the play of power remains beneath the surface, contestation implies open disagreement. An example that is directly linked to *a legal accountability strategy* is the ambiguous working of Liberia’s post-conflict Truth and Reconciliation Commission (TRC). Despite the TRC’s recommendations that 51 individuals, including serving government officials be banned from holding office because of serious allegations or evidence of crimes, including sexual violence, they had committed or supported during the civil war, the accused men called a press conference and warned that Liberia would return to a state of war. Several members of the TRC received death threats and two went into hiding [46]. The constitution of a domestic-international court or what Human Rights Watch termed “hybrid international-national accountability mechanism” did not seem to diminish the distrust of the TRC report, including even by its natural allies. The rejection of the TRC report both by those indicted in it and those running the government, ensured that none of its recommendations could be legally enforced, though the TRC was supported by ordinary Liberian citizens. While formal amnesty could not be written into the text because that would alienate various civil society organizations, and would also be an admission of guilt, the ambiguity around prosecution with much that was left unsaid, coupled with the collapse of the Liberian criminal justice system, translated into de-facto amnesty for the perpetrators and did not yield a single prosecution. The continuing power of high-level military personnel seemed to overwhelm the social mobilization that had led to the ending of conflict [47].

#### Negotiation

Negotiation may follow contestation to explicitly alter an accountability strategy. In contrast to Liberia, the continuing power of former combatants and perpetrators of sexual violence in conflict was better negotiated in Colombia. Colombia’s Law 975, known as the Justice and Peace Law attempted to reintegrate former paramilitaries and ex-combatants into civilian life through a set of measures including reducing their prison sentences. This was opposed by several women’s groups who felt this indirectly offered impunity to those who had perpetrated sexual violence. The Constitutional Court responded to these concerns by directing the government to strengthen reparations by liquidating the assets of the paramilitary to compensate survivors of sexual violence and specific programs to ensure economic and social protection for women, including allocating land titles to displaced women [48]. The subsequent Peace Accord in 2016 also made it explicit that sexual violence was a crime for which there could be no amnesty [48]. The extent to which the government has complied with these commitments is unclear so far, but negotiated *legal accountability* did strive to balance the reintegration of former fighters in order to prevent recidivism with the demands of gender justice and non-impunity for sexual violence.

#### Transformation

We include four illustrations where accountability strategies were able to transform power relations and partially or fully succeed in meeting SRHR objectives including realization of human rights.

Our *first* illustration is the use of Confidential Enquiry into Maternal Deaths (CEMD) in Malaysia. CEMD was introduced after a slew of measures had succeeded in bringing down the Maternal Mortality Ratio (MMR) from 700 per 100,000 live births at independence in 1947 to under 25 in the early 1990s [49]. In order to further reduce the MMR, the CEMD was introduced in 1991 following on high level, multi-stakeholder meetings between the health ministry, obstetricians/gynaecologists and family health officers. CEMDs have been used constructively in a spirit of collaboration, “no-shame, no-blame”, and in a non-punitive way [50]. Improving system functioning was a more important objective than apportioning blame. Smith et al. [51] argue that CEMDs have had special success as a tool for enforcing *performance accountability* because the participants come from the highest levels of the state such as the Ministries of Health as well as multilateral support from the WHO, UNICEF, UNFPA, and national and sub-national professional bodies including the International Federation of Obstetrics and Gynaecology.

A *second* important case of transformation is the change in Ireland’s abortion law as a result of intensive social mobilization. The experience of Ireland which overturned a 35 year ban on abortion in May of 2018 suggests that, through domestic popular mobilization combined with pressure from international bodies, it is possible to counter the power of long entrenched organizations and ideologies and to move towards greater respect for women’s bodily autonomy, and stronger *legal accountability*. In 2012, the avoidable death of a young woman who was refused a life-saving abortion of an unviable fetus despite the fact that the procedure would have been legal even under the strict Irish law of the time, brought thousands of protestors onto the streets of Dublin [52]. In 2018, after considerable ongoing pressure, the rigid Constitutional amendment against abortion was itself overturned. This followed a long process which was set off after the judgment of the court. Importantly, this led to the creation of the Joint Committee on the Eighth Amendment to the Constitution, which recommended full repeal of the Amendment and law reform to legalize abortion on a woman’s request without restriction as to reason within the first 12 weeks of pregnancy, and thereafter in cases where there is a risk to the health or life of the woman [53]. The argument equating the life of the woman with the life of the fetus lost the day. An instructive element in this case is that the demand for legal accountability under an existing restrictive law opened up intense public debate about the human rights basis of the law itself.

Our *third* illustration is about *social accountability* in the context of disrespect and abuse in maternal care [54], which has been termed ‘obstetric violence’ in Latin America. Since the early 1990s in Brazil, social movements played an important role in contesting public policies and legislation, disseminating information to the public, partnering with the Ministry of Health to humanize childbirth, and training healthcare providers [55]. As in many contexts, the behavior and practices of healthcare providers were difficult to change because health workers at the bottom of a power hierarchy are often not free to make positive changes in service delivery [54].

Other pathways to social accountability are described by Hernández and colleagues [56], Schaff and colleagues [57] and by Joshi [58].

Our *fourth* illustration is one of *legal accountability* for respectful maternal care. Successful legal cases in this area are relatively uncommon both because global recognition of the problem of disrespect and abuse of women in obstetric wards has only recently begun to gain momentum, and also because of the “power-knowledge” of health providers. Health providers are experts holding specialized knowledge, with their on-the-spot judgement calls being viewed as needed and legitimate, and rarely challenged. This holds *a fortiori* in the field of obstetrics which is viewed as highly unpredictable. This power has increasingly been challenged through social mobilization, and calls for greater performance accountability [59], but legal cases are relatively rare. The cloak of impunity was lifted recently in a case in Kenya where a woman was abused while in labor in a public hospital, with violations to her dignity and reproductive rights [60]. During the legal case filed on her behalf, the defendants argued that there had been no willful abuse but only the consequences of resource constraints in public hospitals, making respectful care difficult to ensure. The court ruled in favor of the woman, and its “ruling embraced substantive justice over legal procedural technicalities” (60, p125), ordering both an apology and substantial compensation.

### Assessing power in accountability strategies

The illustrative examples above allow us to retrospectively see how power works within accountability for SRHR, but how can we do this prospectively? We propose a power-focused framework - the four sets of questions below - that aims to assess the interactions of different stakeholders with the artefacts of an accountability strategy based on their social position, material interests and voice. These questions can guide realist evaluations [61] of the strategy in terms of its processes / outcomes.

1. Who are the stakeholders in an accountability strategy and what types and sources of power do they wield?
2. What are the different ‘artefacts’ of the accountability strategy (viz., stated objectives, rules, laws, social norms, guidelines, procedures and processes; financing methods and financial/administrative controls)? How do these reinforce or contest the power and/or position of different stakeholders, both duty bearers and rights holders?
3. What incentives and disincentives emerge from the above for different actors? How do different actors respond to these incentives and disincentives, drawing on their sources and types of power? How do power and the artefacts of a strategy shape the values, attitudes and behaviors of different stakeholders?
4. What strategies can be developed that respond to the sources, types and workings of power?

We illustrate how this framework can be used to assess specific questions about the workings of the three accountability strategies we have been addressing, one from each of the categories identified in the systematic review by Van Belle and colleagues [2]: ‘performance’, ‘social’ and ‘legal’ accountability.

#### Illustration of performance accountability: maternal death reviews

Maternal death reviews (MDRs) typically aim to make every pregnancy-related death count by seeking to understand why it occurred and identify steps that must be taken to prevent future recurrences. The creation of such actionable knowledge is a power-filled process. Reviews can take the form of confidential enquiries or death reviews in an area, facility or community.

Decisions about the type of review and of the way it is conducted depend on who is driving the initiative and how they view maternal deaths. For example, the leadership of well-functioning health systems with internal accountability mechanisms may prefer confidential enquiries that emphasize systemic transformation to punitive action against erring health staff. In contrast, civil society organizations seeking retributive justice for maternal deaths resulting from egregious health worker behavior in poorly functioning health systems may prefer community-based death reviews with a strong emphasis on redress and public action.

Each type of review has its own set of rules, procedures and other artefacts, which influence the workings of the strategy. Furthermore, hierarchical power relations between frontline health providers and the communities they serve, as well as within each group (between doctors, nurses, and lower levels of assistants on the one hand, and between community members based on their wealth/income, gender, caste, ethnicity or other ascription on the other) can also have significant impacts on how the strategy is likely to work. These power relations among and between duty bearers and MDR implementers interacting with the strategy’s artefacts drive the processes and quality of data collection and analysis, and can introduce biases in diagnoses of the medical and social causes of death, and in the identification of corrective actions. For example, in our experience of area- or facility-based reviews, lapses by the staff of health facilities are seldom acknowledged as important contributors to preventable mortality, when attending doctors or their peers reconstruct the sequence of events leading to death [62, 63]. The role of power can be fully appreciated by using our power-focused framework to develop a series of specific questions about the MDR. These are listed in Table 1. While the questions are detailed, there may be additional ones that are appropriate for assessing the workings of CEMDs.

**Table 1:** Questions about Maternal Death Reviews

***Types and sources of power among different stakeholders***	
• Which of the duty bearers for maternal safety are officially part of the MDR system? Who is left out? Why?	
o Is the family of the deceased woman represented?	
o Are the attending doctor, nurse and other health providers involved?	
o Do health supervisors or hospital/health system administrators participate?	
o Are external experts (e.g., obstetrician/gynaecologist, anaesthetist) invited?	
o Are government officials (e.g., district commissioner), elected representatives, community health workers, civil society representatives included?	
o What are the implications of their inclusion or exclusion for how knowledge about each death is constructed and for what types of corrective actions are identified?	
• What types of power do each of the duty bearers wield? What are the sources of their power (e.g., access to and control over material resources, knowledge, the bureaucracy, the courts, the police, the media, elected representatives, government officials, or others; influence over decision-making affecting the community and/or the lives of others; social and/or cultural capital)? Which of these individuals have the power to prevent the MDR from achieving its goals?	
• What are the interests of each of the duty bearers who are officially part of the MDR? Are their interests aligned or at odds with each other? What resources can (and do) these individuals galvanise to protect their interests?	
• If any duty bearer is excluded from the MDR, what are his/her interests? Do these individuals try to exert their power over the review and its outcome? When and how do they do so?	
***‘Artefacts’ of the accountability strategy***	
• What are the specific objectives of the MDR? Are the stated objectives to show that there is no impunity for maternal deaths? To prevent recurrence? Or to put systems in place to improve functioning?	
• Who is in charge of the MDR? What is the source of this person’s authority?	
• How is the MDR financed? Are the resources adequate to support the participation of all stakeholders? Who has to sign off on expenses? How soon are payments or reimbursements made? What does this mode of financing imply for the rigour and independence of the review process?	
• How and by whom are pregnancy-related deaths brought to the notice of the health bureaucracy and local administration? How do health managers and local administrators respond officially and unofficially to such deaths? How do communities respond?	
• Which of the pregnancy-related deaths occurring in an area are reviewed? Those occurring within or outside a healthcare facility? Those occurring while women are being taken from one facility to the other?	
• What instrument(s) are used to gather clinical data and information about the sequence of events leading to death? How comprehensive are these instruments? What biases are likely to creep in due to missing or partial questions?	
***Artefacts’ of the accountability strategy (continued)***	
• How and by whom is information recorded in the MDR instrument(s)? Are there safeguards against misrepresentation of the facts by duty bearers who either have vested interests or have ended up contributing to the death? If so, what? What is the quality of the information that is gathered?	
• Who analyses the information gathered through the MDR instrument(s)? Are all sources of information considered, and is a 360-degree approach used? If not, how is the quality of the analytical outputs emerging from this exercise likely to be affected? (e.g., verdicts on the medical cause(s) of death, social and/or health system factors contributing to death)	
• How, where and by whom are the MDR results reviewed? In what spirit are the reviews conducted? How are medical errors and familial failures viewed by reviewer(s)? What responses do they typically evoke?	
• Are corrective actions identified for the health system and the community? How and by whom? What types of actions have tended to be identified?	
• How does the MDR reinforce or contest the power and/or position of individuals who bear the biggest responsibility for maternal safety?	
***Incentives and disincentives to different actors and their resulting behaviour patterns***	
• What do family members as well as attending doctors, nurses and other health staff stand to lose if they are implicated in the death?	
• How do they respond to real or feared penalties that are meted out to “guilty parties” as part of the MDRs? (e.g., by misrepresenting facts; preventing others from reporting information; doctoring records to indicate causes of death that are unpreventable, etc.)? How is such behaviour justified?	
• What are the implications of a maternal death for the health care facility’s leadership (e.g., drop in the facility’s rating; scrutiny by peers or superiors in the health bureaucracy; loss of face among peers; no difference; etc.)?	
• How do these leaders respond to other obstetric emergencies occurring in their facilities (e.g., not admitting women in need of care that can be provided by the facility; referrals to ensure that women don’t die in their facility, etc.)? How is such behaviour justified?	
• Are there any incentives or disincentives for family members, attending health providers and other individuals who were directly involved to provide complete information about the death, as they know it, and to willingly participate in the MDR? If so, what?	
***Consequences for the accountability strategy***	
• Do MDRs fairly recreate the sequence of events leading to death and to what extent? Why and how?	
• Do MDRs allow health systems and communities to learn from and become more accountable for preventable maternal mortality and to what extent? Why and how?	
• Do MDRs provide redress to families of the women whose deaths could have been prevented? Why, how and to what extent?	

#### Illustration for social accountability: community-based monitoring

At its core, community-based monitoring (CBM) is intended to reverse one-sided and top-down relationships between public service providers, related functionaries and communities. The strategy generally aims to make state-run health services cognizant of and responsive to the priorities, needs and human rights of people in communities. These goals can only be achieved if the cultures of service delivery systems are challenged. As Fox [64] points out, the impacts of social accountability initiatives depend on whether there is an enabling environment for collective action along with state capacity to respond to citizens, pointing to the need for greater state-society synergy. We believe such synergy is predicated on power relations. Thus, CBM is a highly political strategy that can be more fully understood if the workings of power are unpacked. We use our framework to guide specific questions that can be asked, which are listed in Table 2.

**Table 2:** Questions about Community-based monitoring

***Types and sources of power among different stakeholders***	
• Who determines the nature of participation by the community? What are the costs and benefits of participation? To what extent does the community itself determine the objectives and methods of the strategy?	
• Which sections of the community are included? Who is left out?	
o Are community leaders involved? Who are they (e.g., elected representatives, government officials, civil society leaders, thought leaders, large landowners/ entrepreneurs employing labour, other leaders, etc.)?	
o Are all sub-groups adequately represented across, inter alia*,* the gender, age, ability and socioeconomic spectrum as well as ethnic and religious groups? If some sections of the community are under- or unrepresented, why is this so?	
o What are the implications of their inclusion or exclusion for the workings of the strategy?	
o What does representation imply in terms of responsibility and transparency? Voice? Ability to demand and obtain services?	
• What types and sources of power do each of the stakeholder groups wield (e.g., access to and control over material resources, knowledge, the bureaucracy, the courts, the police, the media, elected representatives, government officials, etc.; influence over decision making affecting the community and/or the lives of others; social and/or cultural capital)?	
• Which of these individuals or groups have the power to prevent the strategy from achieving its goals? What aspects of their power must be brought to book? Their ownership and/or control over material resources? The scope of their influence over major decisions affecting the lives of others and the achievement of collective goals? The dominance of their voice and/or their authority in the community?	
• What are the interests of each of the stakeholders who are formally part of the CBM exercise? Are their interests aligned or at odds with each other? What resources can (and do) these stakeholders galvanise to protect their interests?	
• What are the interests of the stakeholders who are left out of the CBM exercise? Do these stakeholders try to influence the monitoring process and its outcome? When do they do so?	
***Accountability ‘artefacts’ in the strategy***	
• What are the goals of CBM? Who sets these goals for the community?	
• Overall, who is responsible for implementing the strategy? What is the relationship between this individual’s office and the community?	
• What does the CBM work cycle look like? What structures (e.g., committees, groups, etc.), processes (e.g., data collection, consultations) and instruments (e.g., scorecards, etc.) are developed as part of the strategy? Which sections of the community have voice and say in the strategy’s structures, processes, instruments and work cycles? Which sections have neither voice nor say?	
***Accountability ‘artefacts’ in the strategy (continued)***	
• How, where and by whom is information about community needs, service uptake and health outcomes (among others) gathered? Are there safeguards against misrepresentation of the facts by stakeholders who have vested interests and the power to influence others? What safeguards are these? What is the quality of the information that is gathered?	
• Who analyses the information gathered through the strategy’s structures, process, and instrument(s)? Do analytical outputs emerge from this exercise? Are these outputs reviewed and/or validated by different stakeholders/sections of society?	
• Are action plans identified based on analytical inputs? How and by whom? What types of actions?	
• How are groups that are resistant to the goals of CBM viewed by the individuals leading the effort? How are such groups handled (e.g., censured, isolated, stigmatised at one extreme to consulted to modify the strategy at the other)?	
• Are there adequate redressal mechanisms for those who are disempowered and/or adversely affected by abuses of power?	
• Who finances the CBM exercise? What does the mode of financing imply for the independence of the strategy and for the autonomy of participating stakeholders?	
***Incentives and disincentives to different actors and their resulting behaviour patterns***	
• Do different stakeholders stand to gain or lose from the strategy? If so, what?	
• How do stakeholders respond to real or feared disempowerment due to the strategy?	
• How often and among which stakeholders are these behaviours to be found? How are such behaviours justified?	
***Consequences for the accountability strategy***	
• What happens when controversial issues arise? Whose views prevail?	
• Who benefits and who loses from the strategy and its instrument(s)? Where? When? How? Why?	
• Who remains untouched by the strategy? How? Why?	
• Are relationships between the powerful and those who are disempowered different since the accountability strategy was introduced? How? With what effect? Why?	
• What contextual factors (moral and political economies, gender norms, and challenges linked to the claiming of rights) contribute to or prevent changed relationships?	

#### Illustration for legal accountability

Of the three types of accountability strategies considered, legal accountability is the one with the clearest standards against which accountability can be measured. National Constitutions and laws together with international covenants, treaties and agreements provide a framework of norms against which violations of SRHR can be compared. Table 3 provides the questions to guide such comparisons.

**Table 3:** Questions to elicit the working of legal accountability

***Identifying which sexual or reproductive rights have been violated***	
• Identify the elements of the rights violated and identify the problems that led to the violation	
• Identify the factors that led to the victim’s mortality or morbidity e.g. age, existing conditions; knowledge of rights; access to services; availability of services needed at the facility	
***Responsibilities of the duty bearer***	
• Assess whether the government (identify which body of the government in particular) may be accused of failing to:	
o provide the required services;	
o attend to the underlying determinants of health;	
o invest in programs to raise awareness of sexual and reproductive health rights;	
o take concrete, targeted steps to realise the right;	
o provide information and opportunities for residents to participate in decision-making regarding the quality and provision of services; and	
o uphold other human rights including the right to education.	
***Applicable national laws and policies***	
• Do the policies show how the government is planning to extend health care to all areas?	
• What information about SRHR services and disease burden is available?	
• Are there statistics relating to the district or local levels?	
• Does this information show discrimination in favour of cities or areas where richer people live?	
• What are the main causes of SRHR related mortality reported in the census or demographic health data?	
• Does the government have plans and projects to deal with these?	
• What is the condition of SRHR services in the area where the victim resides?	
• Are they adequately staffed and equipped?	
• Is there a system for making a complaint about health services?	
• What has the government done to provide or improve SRH services?	
***Actions or omissions violating SRHR***	
• What national law (if any) has been broken and how?	
• What regional and international standards apply?	
• Which human rights obligations has the government failed to carry out?	
• Under which article of the law or treaty?	
Refer, as appropriate, to General Comment 14, 22 or other sources or to relevant decisions of national courts or accountability mechanisms.	

### Summing up

This paper addresses the challenge of power in accountability strategies to realize SRHR. It draws on recent and more classic streams of literature that address frameworks of power and empowerment; power in the context of health policy processes; and accountability strategies for SRHR. It is premised on the understanding that SRHR are embedded in power relations that shape the realization of human rights. Hence power must be central to how we think about and assess accountability strategies.

The paper develops a synthetic framework of types, sources and workings of power. This synthesis advances recent work on power in health by recognizing the combined importance of structural including ascriptive inequalities on the one hand, and beliefs and norms on the other. The paper also reflects on the implications of power’s capillarity to further our understanding of how power works. We have argued that, for accountability strategies, power may work in six different ways – reinforcing dominance, accommodation, subversion, contestation, negotiation and transformation. These workings are illustrated through brief cases of accountability strategies for different elements of SRHR.

The paper goes on to draw out the implications of the above analysis for prospective assessment of accountability strategies for SRHR. We argue that power-focused realist evaluations are needed using the four sets of questions defining our framework, about: i) the dimensions and sources of power that an accountability strategy confronts; ii) how power is built into the artefacts of the strategy – its objectives, rules, procedures, financing methods inter alia; iii) what incentives, disincentives and norms for behavior are set up by the interplay of the above; and iv) their consequences for the processes and outcomes of the accountability strategy. We illustrate this approach through examples of performance, social and legal accountability.

Our approach provides a nuanced way to understand how power shapes accountability strategies and our assessment of them. It also opens the door for thinking more systematically about policy elements that may need to be built into accountability strategies for SRHR (and for health more broadly) that recognize and address the role of power. These include not only actions that may confront the powerful directly, but also other ways to strengthen subordinated and oppressed people to realize their human rights. Such actions can include, among others, adapting financing, rules, procedures and guidelines to be friendlier to girls and women; building the capacity of communities and of girls and women within them to contest, negotiate with, or subvert dominant power relations; supporting civil society to be interlocutors for accountability (not service providers); and intervening on what data are collected and how, and how data are used to advance accountability for health and human rights.

## Data Availability

Data sharing not applicable to this article as no datasets were generated or analysed during the current study.
